# Hemangiopericytoma of the spleen: Unusual presentation as multiple abscess

**DOI:** 10.1186/1477-7819-3-77

**Published:** 2005-12-17

**Authors:** Mukesh Kumar, Kamlakar Tripathi, Rahul Khanna, Mohan Kumar, Ajay K Khanna

**Affiliations:** 1 Department of General Medicine, Institute of Medical Sciences, Banaras Hindu University, Varanasi, India; 2 Department of General Surgery, Institute of Medical Sciences, Banaras Hindu University, Varanasi, India; 3 Department of Pathology, Institute of Medical Sciences, Banaras Hindu University, Varanasi, India

## Abstract

**Background:**

Hemangiopericytoma is a soft tissue vascular neoplasm arising from capillary pericytes and is found throughout the body in soft tissues and bone. It was first described in 1942. Primary vascular neoplasm of the spleen constitutes the majority of nonhaematolymphoid splenic tumors like haemangioma, lymphangioma, hemangioendothelioma, hemangiopericytoma etc. Splenic hemangiopericytoma is a rare tumor and probably first case was described in 1989. Uptill now only eight cases are reported in the English literature.

**Case presentation:**

A-35-year old male presented with fever and dull aching pain in left hypochondriac region. Radiological evaluation showed presence of multiple abscesses in spleen. Investigations were done to rule out common causes of abscess in spleen. After failure of medical management, he was subjected to elective splenectomy. There were dense adhesions between the spleen and the adjacent structures and the diaphragm. The histopathology of the resected specimen showed hemangiopericytoma of spleen.

**Conclusion:**

The present case illustrate that the hemangiopericytoma of spleen can mimic as multiple abscess. Splenectomy is the treatment of choice.

## Background

Hemangiopericytoman is a soft tissue vascular neoplasm arising from capillary pericytes and is found throughout the body in soft tissues and bone [1-3]. It was first described in 1942 [1]. Primary vascular neoplasm of the spleen constitutes the majority of nonhaematolymphoid splenic tumors [4]. The benign vascular tumors include haemangioma, hamartoma and lymphangioma whereas those of variable or uncertain biologic behavior include littoral cell angioma, hemangioendothelioma and hemangiopericytoma. They are mostly seen in extremities but may be present in retroperitoneum, head and neck, chest and abdomen [2,5]. Splenic hemangiopericytoma is a rare tumor and probably first case was described in 1989 by Guadalajara *et al*, and so far only eight patients have been reported in the literature [4,6-13]. It tends to occur at adult ages, but can be diagnosed in childhood [6]. The presentation of splenic tumor is variable because of pain or rupture or lump or hemorrhage but presentation as splenic abscess has not been described [7-13]. Yilmazlar *et al*, recently, described a case of Splenic hemangiopericytoma and serosal cavernous hemangiomatosis of the adjacent colon and suggested a sequential relationship [12].

## Case presentation

A 35-year-old male presented with complaint of mild grade, intermittent fever without chills and rigor and diurnal variation for last 2 months along with occasional dull aching pain in left hypochondriac region. Ultrasonography revealed multiple abscesses in spleen. He was managed on antibiotics for 21 days but fever continued. Computed tomographic (CT) scan of abdomen showed multiple abscesses in spleen without any other significant findings in abdominal organs. Around 200 ml of pus was aspirated under guidance, which was negative for bacteriological examination. He was kept on parenteral antibiotics but did not have any relief.

Color Doppler of heart excluded any possibility of infective endocarditis. X-ray chest was normal. Montoux test was positive up to 11 × 11 mm and he was put on antitubercular treatment to which he did not respond in 6 weeks. Surgical consultation was done and it was decided to proceed with splenectomy.

At laparotomy, there were dense adhesions between the spleen and the adjacent structures and the diaphragm. The patient went into shock after 2 hours of surgery and was resuscitated and taken back to operation theatre for reexploration. Exploration revealed generalized ooze from the area and hemostasis was achieved but again after 2 hours, the patient went into shock and was reexplored again but the patient succumbed to the continued hemorrhage.

**Figure 1:**
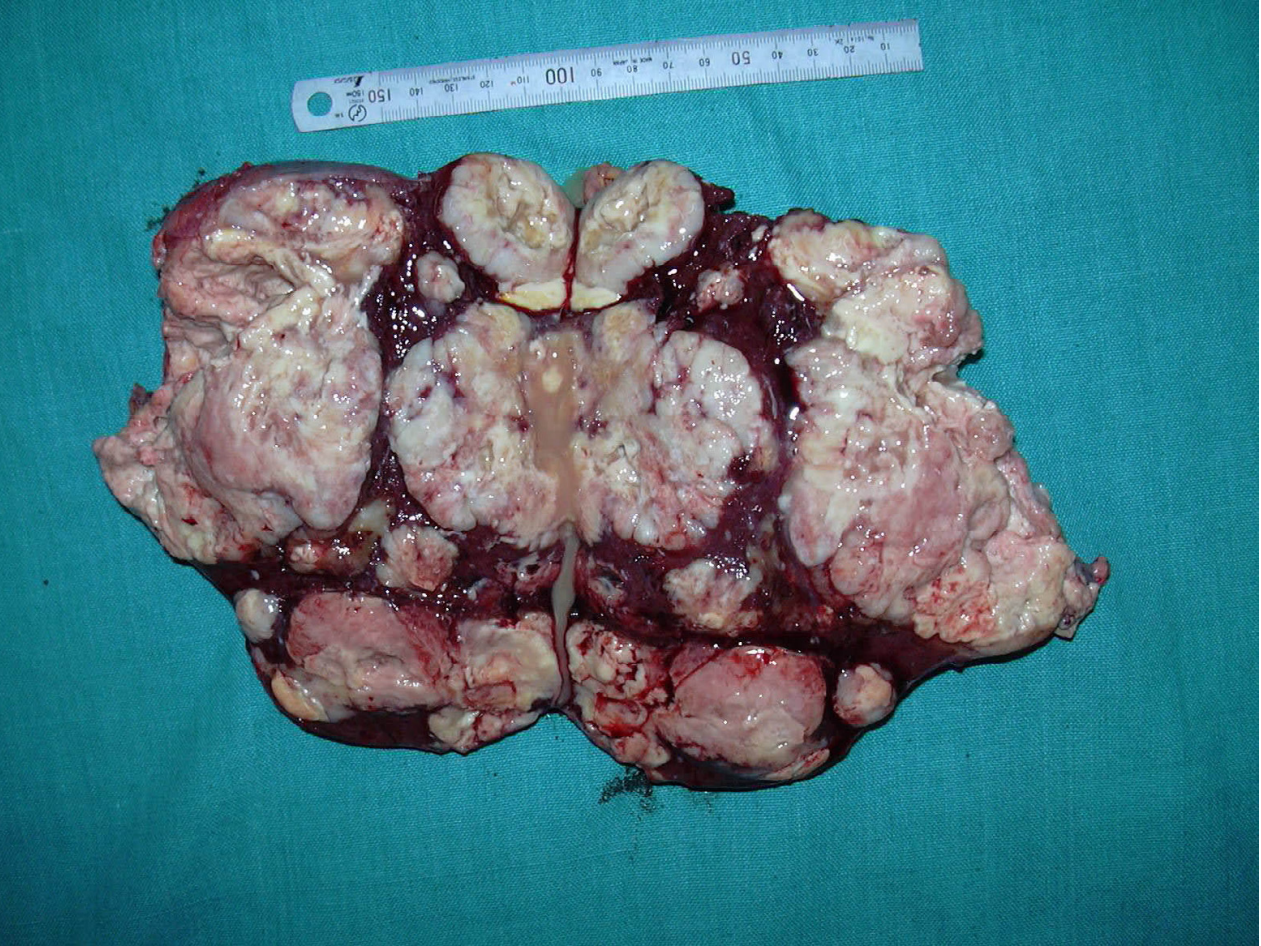
Resected specimen measured 18 × 12 × 6 cm and cut surface showed multiple lobulated, solid, well circumscribed nodules of variable sizes 1 cm to 7 cm in diameter with areas of hemorrhage and cystic degeneration.

**Figure 2:**
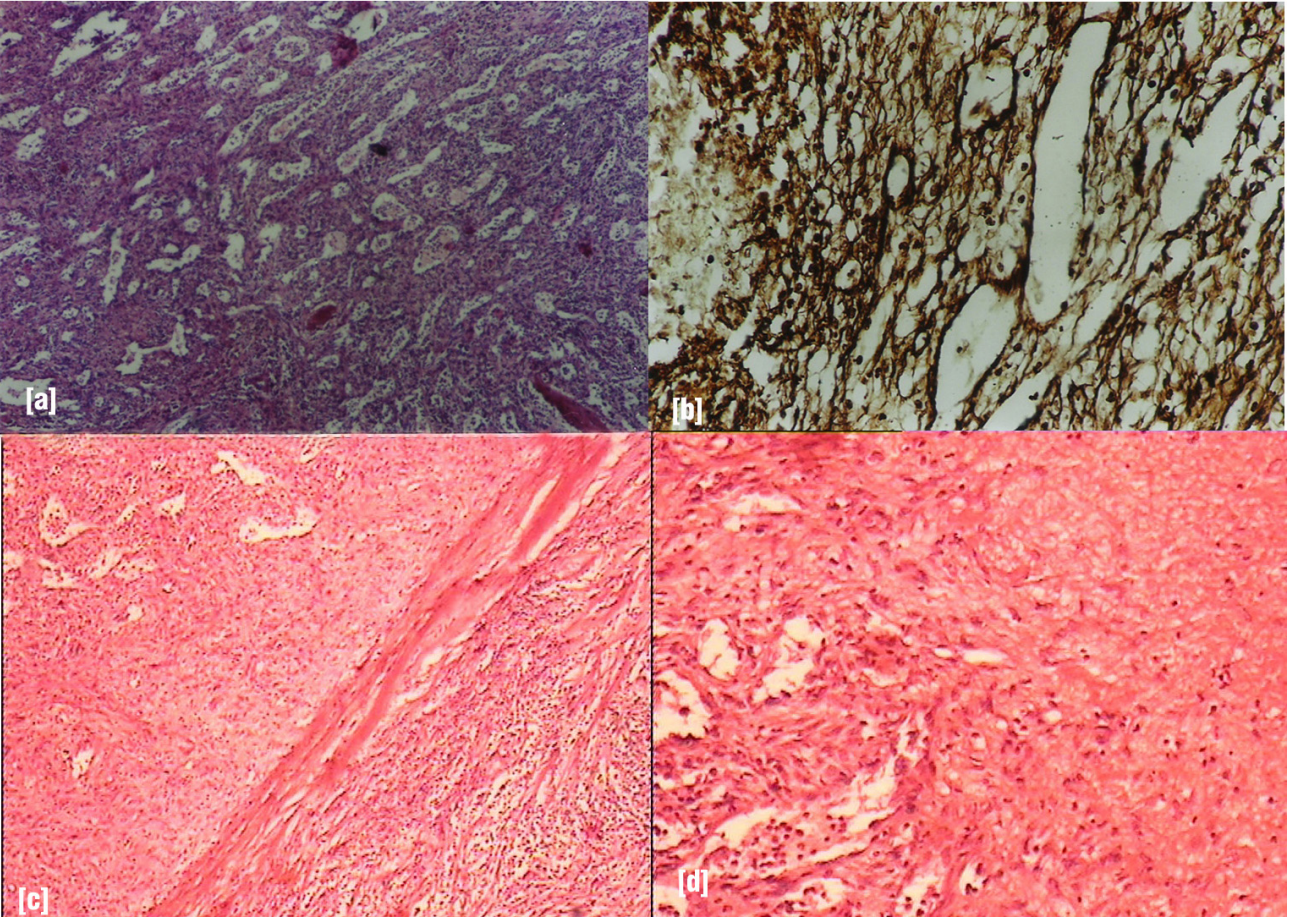
(a) shows dilated, sinuous vascular channels surrounded by oval to spindle tumor cells, having scanty cytoplasm and hyperchromatic nuclei, mitotic figures occasional present (Hematoxylin & Eosin ×200); (b) Reticulin stain (×200) shows intricate reticulum laid down around tumor cells and dilated vascular channels; (c) & (d) shows fibrous capsule of the tumor along with areas of necrosis (Hematoxylin & Eosin ×50).

Gross specimen measured 18 × 12 × 6 cm and cut surface showed multiple lobulated, solid, well-circumscribed nodules of variable sizes 1 cm to 7 cm diameter with areas of hemorrhage and cystic degeneration replacing most of splenic parenchyma (Figure 1). The microscopic examination on Hematoxylin & Eosin stain showed dilated, sinuous vascular channels surrounded by oval to spindle tumor cells having scanty cytoplasm and hyperchromatic nuclei, mitotic figures were occasional (Figure 2). There were areas of hemorrhage and cystic degeneration. The Reticulin stain showed intricate reticulum laid down around tumor cells and dilated vascular channels suggestive of hemangiopericytoma (Figure 2).

## Discussion

Primary vascular neoplasms of spleen constitute the majority of nonhematolymphoid splenic tumors. The benign primary vascular tumors include hemangioma, hamartoma and lymphangioma whereas those of variable or uncertain biologic behavior include littoral cell angioma, hemangioendothelioma and hemangiopericytoma [4]. Despite the advances in imaging techniques, splenectomy may be required for definitive evaluation of splenic abnormality with atypical features. In the present case, radiological investigation did not suggest possibility of hemangiopericytoma or least a malignancy. In most of the cases, splenectomy seems to be necessary for establishing prompt histologic diagnosis and providing initial treatment of the disease. Initially in our patient we suspected infective causes of splenic abscess but normal cardiological examination and failure of response to medical management lead to a possibility of non infective cause of splenomegaly and that is why the patient was subjected for surgery.

Most hemangiopericytoma om cytogenetic analysis show near near-diploid and breakpoints in 12q13, 12q 24 and 19q13 seem to be common with t (12;19)(q13;q13) being a recurrent translocation. Hallen suggested that karyotypic pattern of hemangiopericytoma of spleen may differ from hemangiopericytoma of soft tissue [14]. It is difficult to differentiate whether splenic hemangiopericytoma was benign or malignant. It was suggested that presence of multifocal tumors or mitoses might be an indicator of poor prognosis. Yilmazlar refute the possibility of multifocal tumors as predictor of malignant tumor behavior [12]. Thus, it is too early to say that a particular feature will define benign or malignant behavior meanwhile presence of mitoses is suggested factor. Hemangiopericytoma of spleen was first reported in 1989 and second one in 1992 of multiple hemangiopericytoma of spleen [9,11] One of the case reports of hemangiopericytoma of spleen in 1997 presented as recurrent massive upper gastrointestinal bleeding [13]. Yilmazlar described a case of hemangiopericytoma of the spleen and serosal cavernous hemangiomatosis of the adjacent colon and suggesting a sequential relationship [12]. This is probably, the first ever case report of hemangiopericytoma of spleen presenting as multiple abscesses in the English literature. Treatment of choice in all cases remains splenectomy with the addition of radiation or chemotherapy in selected cases.

## Conclusion

This case is of multiple abscesses of spleen showing unusual histopathology. Till date, hemangiopericytoma of spleen was described with recurrent massive upper gastrointestinal bleeding or serosal cavernous hemangiomatosis of the adjacent colon or pain or rupture or lump but no case was reported with presentation as multiple abscesses in spleen. [6-13].

## Competing interests

The author(s) declare that they have no competing interests.

## Authors' contributions

All the authors equally participated in preparation of the manuscript.

## Funding source

None
